# Stroke severity to determine musculoskeletal symptoms in family caregivers

**DOI:** 10.1590/1518-8345.6725.4005

**Published:** 2023-10-09

**Authors:** Tugba Sahbaz, Cansın Medin-Ceylan

**Affiliations:** 1 Beykent University, Faculty of Medicine, Department of Physical Medicine and Rehabilitation, Istanbul, Turkey; 2 University of Health Sciences, Istanbul Physical Therapy Rehabilitation Training and Research Hospital, Department of Physical Medicine and Rehabilitation, Istanbul, Turkey.

**Keywords:** Back Pain, Caregivers, Disability Evaluation, Pain, Musculoskeletal Pain, Stroke, Dolor de Espalda, Cuidadores, Evaluación de la Discapacidad, Dolor, Dolor Musculoesquelético, Accidente Cerebrovascular, Dor nas Costas, Cuidadores, Avaliação da Deficiência, Dor, Dor Musculoesquelética, Acidente Vascular Cerebral

## Abstract

**Objective:**

the objective of this study is to examine the relationship between the musculoskeletal problems experienced by the family members who care for stroke patients, physical health and disability levels.

**Method:**

the subjects included in the study were patients and family caregivers admitted to the Kanuni Sultan Suleyman Training and Research Hospital Physical Medicine and Rehabilitation outpatient clinic with a stroke diagnosis between May 30 ^th^, 2019, and May 30 ^th^, 2021. The caregivers were assessed using the Extended Nordic Musculoskeletal Questionnaire. Validated scales were employed to evaluate stroke patients’ physical health and disability level.

**Results:**

a total of 104 stroke patients and 104 caregivers who met our inclusion criteria took part in this study. Low back complaints in the last month were associated with the patients’ Functional Ambulation Score (FAS), Functional Independence Measure (FIM), Stroke Impact Scale (SIS) and Brunnstrom scores. Neck pain was the second musculoskeletal complaint, but was not statistically associated with patient-related factors. Upper limb problems were associated with FAS, FIM, SIS, Brunnstrom and the Modified Ashworth Scale scores.

**Conclusion:**

according to our findings, the low back is the body area most affected by musculoskeletal complaints in family caregivers of stroke patients, closely related to the patients’ functional capacity and disability levels. Clinical trials number: NCT04901637

## Abstract (es)

**Objetivo:**

el objetivo de este estudio es examinar la relación entre los problemas osteomusculares sufridos por cuidadores familiares que tienen a su cargo velar por pacientes que han sufrido un accidente cerebrovascular y los niveles de salud física y discapacidad.

**Método:**

los sujetos incluidos en el estudio eran pacientes y cuidadores familiares atendidos en la clínica de servicios ambulatorios de Fisioterapia y Rehabilitación del Hospital Universitario y de Investigación Kanuni Sultan Suleyman por diagnósticos de ACV entre el 30 de mayo de 2019 y el 30 de mayo de 2021. Los cuidadores fueron evaluados mediante el *Extended Nordic Musculoskeletal Questionnaire*. Se emplearon escalas validadas para evaluar los niveles de salud física y discapacidad de los sobrevivientes de accidentes cerebrovasculares.

**Resultados:**

los participantes de este estudio fueron 104 sobrevivientes de accidentes cerebrovasculares y 104 cuidadores que cumplieron con nuestros criterios de inclusión. Las quejas relacionadas con la región lumbar durante el último mes se asociaron con las puntuaciones obtenidas por los pacientes en el *Functional Ambulation Score* (FAS), la *Functional Independence Measure* (FIM) y la *Stroke Impact Scale* (SIS), además de las puntuaciones Brunnstrom. El dolor de cuello fue la segunda queja osteomuscular, aunque no se asoció estadísticamente con factores relacionados con los pacientes. Los problemas en las extremidades superiores se asociaron con las puntuaciones obtenidas en los instrumentos FAS, FIM, SIS, Brunnstrom y *Modified Ashworth Scale*.

**Conclusión:**

de acuerdo con nuestros hallazgos, la región lumbar es la parte del cuerpo más afectada por quejas osteomusculares en cuidadores familiares de sobrevivientes de accidentes cerebrovasculares, demostrando una estrecha relación con los niveles de capacidad funcional y discapacidad de los pacientes. Número de registro de ensayos clínicos: NCT04901637

## Abstract (pt)

**Objetivo:**

o objetivo deste estudo é examinar a relação entre os problemas musculoesqueléticos vivenciados pelos familiares que cuidam de pacientes com AVC, a saúde física e o nível de deficiência do paciente.

**Método:**

foram incluídos no estudo pacientes e familiares cuidadores admitidos no hospital Kanuni Sultan Suleyman com diagnóstico de AVC entre 30 de maio de 2019 e 30 de maio de 2021. Os cuidadores foram avaliados utilizando o questionário *Extended Nordic Musculoskeletal Questionnaire*. Escalas validadas foram usadas para avaliar a saúde física e o grau de incapacidade dos pacientes com AVC.

**Resultados:**

um total de 104 pacientes com AVC e 104 cuidadores atenderam aos critérios de inclusão do estudo. As queixas lombares no último mês foram associadas aos escores do *Functional Ambulation Score* (FAS), *Functional Independence Measure* (FIM), *Stroke Impact Scale* (SIS) e teste de Brunnstrom do paciente. A dor no pescoço foi a segunda queixa musculoesquelética, mas não foi estatisticamente associada a fatores relacionados ao paciente. Os problemas nas extremidades superiores foram associados aos escores FAS, FIM, SIS, Brunnstrom e à *Modified Ashworth Scale*.

**Conclusão:**

e acordo com os nossos achados, a região lombar é a área do corpo mais afetada por queixas musculoesqueléticas nos cuidadores familiares de pacientes com AVC, que estão intimamente relacionadas ao nível de capacidade funcional e ao grau de incapacidade dos pacientes. Número do estudo clínico: NCT04901637.

**Highlights:**

**(1)** Stroke survivors highly depend on informal caregivers for daily living.

**(2)** Family caregivers are at an increased risk of experiencing musculoskeletal problems.

**(3)** The caregivers’ musculoskeletal symptoms are related to the level of the patient disability.

**(4)** Preventive medicine should become a part of nursing education for family caregivers.

## Introduction

Stroke is one of the main causes of neurological disability in individuals at the global level and significantly increases morbidity and mortality, particularly in non-developed and developing nations ^(^ 1 ^-^ 2 ^)^. Yet, the overall prognosis of stroke patients has not improved accordingly, and many patients live with various forms of disability worldwide; there are 15 million cases of stroke each year, of which 5 million require ongoing care due to severe disability ^(^ 2 ^)^. 

Stroke patients typically have varying disability degrees. They need immediate care in a hospital setting and considerable support while recovering at their homes. Most stroke patients rely on unpaid informal caregivers, typically members of the patients’ family (e.g., spouses). Caregivers are frequently unprepared and unsuited to provide such assistance after discharge ^(^ 3 ^)^. As a result, a decrease in levels of general well-being, social life and physical and mental health of individuals can be observed ^(^ 4 ^)^. 

It is noted that Turkey has limited access to caregiving support services and related facilities, including adult daycare and stroke support groups. In addition, close family members typically care for the sick and disabled in our society. These caregivers may receive a pension from the patients, but rarely hire professionals ^(^ 5 ^)^. 

Examining the effects on caregivers is crucial, given how demanding such care is for a person with cognitive impairment ^(^ 6 ^)^. Much research has been undertaken to examine the impact of caregiving with several dimensions, e.g., caregivers’ stress, strain, burden and quality of life (QoL), as stroke caregivers play such a dominant role ^(^ 7 ^)^. 

Several studies have been conducted on subjects, despite the possibility that family caregivers may also be at risk of developing musculoskeletal problems ^(^ 8 ^-^ 9 ^)^. There is wealth of information on physically taxing activities that wear down seasoned caregivers. One of the main causes of musculoskeletal complaints in medical professionals and caregivers is manual lifting of patients, which strains the spine ligaments, particularly the lumbar region. Typically, the shoulder joints, cervical spine and low back are the most impacted body areas ^(^ 10 ^)^. 

The family members, who take care of patients with stroke are at a higher risk of musculoskeletal problems than rehabilitation specialists, given the number of hours devoted to various caregiving activities, with the possibility that they may lack patient handling skills and necessary equipment ^(^ 7 ^)^. 

The objective of the current study was to evaluate musculoskeletal issues that family caregivers experience in connection with the patients, who present physical health problems and/or also disability. In this sense, this is the first study that evaluates the symptoms of the caregivers from their own perspective, comparing severity of their symptoms and also with the level of the patient disability.

## Method

### Study design

In this cross-sectional study, the physical health and disability levels of stroke patients and the musculoskeletal system problems of family caregivers were evaluated with face-to-face survey data to determine the musculoskeletal problems experienced by family caregivers who care for stroke patients. These data were collected for this study.

### Setting

This cross-sectional study was conducted between May 2019 and May 2021 in the Kanuni Sultan Suleyman Training and Research Hospital Physical Medicine and Rehabilitation outpatient clinic. Between these dates, stroke patients who applied to the service for the first time or were followed-up in our clinic and those who provided primary care to these patients at their homes were included in the study. In cases where there was more than one home caregiver, the care time was used to select the primary caregiver. Caregivers performing the role for more than eight hours a day or for over six months were considered as primary caregivers and were included in the study. Consent was obtained both from the stroke patients and from the primary caregivers before the study. All the scales used in the study were filled in face-to-face by a Physical Medicine and Rehabilitation specialist.

### Participants

The patients included were those who suffered a stroke as *per* the WHO definition ^(^ 11 ^)^, who required assistance with activities of daily living (ADLs), were older than 18 years of age, and were considered eligible. Patients who did not require supervision or assistance in activities of daily living, with cognitive inability to understand the test instructions (Mini Mental State Examination < 18), presence of aphasia that precluded communication, and high motor recovery were not included in the study ^(^ 12 ^)^. Patients living in nursing homes were excluded from the study. 

The study included family caregivers over the age of 18 who assisted with basic ADLs and cared for the stroke patients during more than eight hours a day for at least six months. The study excluded caregivers who had been diagnosed any musculoskeletal disease (moderate/severe osteoarthritis, disc herniation, spondylolisthesis, scoliosis, spondylosis, meniscopathy, chronic tendinopathy) before initiating patient care, as well as previous musculoskeletal surgery, neurological disabilities or psychological problems that might limit their ability to provide adequate care. Caregivers older than 65 years of age were also excluded due to the detrimental effects of aging on the musculoskeletal system.

### Study population and sample design

Among the 416 hemiplegic patients who applied to our clinic between May 2019 and May 2021, 51 of them were excluded from the study because of stroke diagnosis less than 6 months ago, 44 due to aphasia, 93 due to cognitive disability, 32 for not having a primary caregiver, and 23 for staying in a nursing home. Among the caregivers of the remaining 177 patients with hemiplegia, 46 were excluded from the study due to musculoskeletal disease, 14 due to psychological problems (use of antidepressant medications), 9 due to previous musculoskeletal surgery, and 4 due to neurologic disabilities.

### Study variables

Demographic data from the caregivers such as age, gender, schooling level, employment status and the Extended Nordic Musculoskeletal Questionnaire (NMQ-E), Caregiver Burden Inventory (CBI) and Beck Depression Inventory (BDI) questionnaire data were asked. The Functional Independence Scale, Stroke Impact Scale, Modified Ashworth Scale, Brunnstrom and Functional Ambulation Score tools were used to determine the stroke patients’ physical and disability status. To assess the relationship between the stroke survivors’ physical and disability status and the family caregivers’ musculoskeletal problems, the caregivers’ extended scores and their own BDS and CBI levels were compared, in addition to evaluating the correlation between the stroke patients’ disability levels.

NMQ-E, a one-page survey for nine body areas (hand/wrist, elbow, shoulder, neck, upper back, low back, hip/thigh, knee and foot/ankle) assessed the impact of musculoskeletal complaints in the previous six months. “Trouble, including aching, agony, and discomfort” was given as an answer. They had both neuropathic and stomach pain; however, only musculoskeletal symptoms (joints, muscles and bones) were assessed ^(^ 13 ^-^ 14 ^)^. A CBI validated version was used to measure perceived care burden. Time dependence, developmental, physical, social and emotional demands were its five areas, and a self-applied 24-item questionnaire was used. On a five-point Likert scale, 0 represented the least disruptive (not at all disruptive) and 4 represented the most disruptive (very disruptive). Combining the subscale scores, the overall score was also determined. Higher values indicated heavier strain on the caregivers ^(^ 15 ^)^. Depression was measured with BDS, which was validated and verified as reliable in Turkey. With 21 items, its scores ranged from 0 to 3. The maximum score was 63, denoting severe depression ^(^ 16 ^-^ 17 ^)^. 

The Functional Independence Measure (FIM) assessed severity of the disability: there were 18 measures for how well a person communicates, takes care of themselves, thinks socially, moves, transfers and controls sphincter issues ^(^ 18 ^)^. With an overall score, a specific motor function score was also provided. This study used a stroke-specific outcome-measuring instrument created by Duncan, et al. ^(^ 19 ^)^. The most recent version of the Stroke Impact Scale (SIS) has 59 items to cover 8 domains, namely: mobility, hand function, strength, ADLs and instrumental ADLs, mood, communication, social involvement, and memory. Each domain was scored from 0 to 100; high total scores indicated acceptable functional recovery ^(^ 19 ^)^. Turkish validity and reliability studies were available for all questionnaires applied to stroke patients and family caregivers ^(^ 11 ^-^ 19 ^)^. 

Upper and lower limb muscle spasticity was measured with the Modified Ashworth Scale (MAS), along with a 6-point scale. The maximum score, 4, shows that the affected limb is rigid during flexion or extension. The lowest score, 0, denotes “no increase in muscle tone” ^(^ 20 ^)^. The motor and tonus analysis, known as the Brunnstrom staging system, evaluated the extent of motor recovery. It established six distinct classes pertaining to the hands and upper and lower limbs. Stage one describes the initial phase, with the least amount of movement, while Stage six describes presence of isolated motion ^(^ 21 ^)^. The patients’ ambulatory status was evaluated with the Functional Ambulation Score (FAS): it used six headings describing the patients’ functional walking. Patients with a score of 0 were unable to walk at all, when compared to those with a score of 5 who ambulated on their own. According to the FAS scale, human aid is preferred to devices and supports ^(^ 22 ^-^ 23 ^)^. 

### Ethical aspects

The 1964 Declaration of Helsinki, as well as any later revisions to it, along with comparable ethical standards, were used in all procedures in this study that involved human subjects. All individuals taking part in the study had given their informed consent.

### Data analysis

The IBM SPSS (Statistical Package for the Social Sciences) software, version 25.0, was used to statistically analyze the study data. Mean and standard deviation (SD) or number and frequency were used to express descriptive data. The Kolmogorov-Smirnov test was used to determine distribution of the variables. The patients were divided into two groups according to presence of pain in the last month for nine body areas. Normal distribution was examined for parametric data, and those with normal distribution were compared with the independent sample t-test for two groups, whereas non-normally distributed parametric data and non-parametric data were compared with the Mann-Whitney U test. Statistical significance was considered as p<0.05.

## Results

A total of 104 stroke patients and 104 caregivers met our inclusion criteria. While 44.2% of the patients were female, 83.7% of the caregivers were also female. Spouses and sons/daughters accounted for 56.7% and 26.9% of the caregivers, respectively. The demographic data from patients and caregivers are presented in Table 1. 

**Table 1 -:** Demographic characteristics of the participants. Istanbul, Turkey, 2019-2021

	**Patients**	**Caregivers**
Gender n (%)		
Female	46(44.2)	87(83.7)
Male	58(55.8)	17(16.3)
Age (Mean ± SD *)	62.83±12.02 (31-87)	51.38±9.80 (27-65)
Affected side n (%)		
Right	59(56.7)	
Left	45(43.3)	
Type of lesion (%)		
Ischemic	83(79.8)	
Hemorrhagic	21(20.2)	
Time since stroke (months)	42.25±42.37	
Time as caregiver (months)		37.27±36.65
Schooling n (%)		
Literate	16(15.4)	19(18.3)
Elementary School	61(58.6)	47(45.2)
High School	14(13.5)	12(11.5)
University	13(12.5)	26(25.0)
Marital status n (%)		
Single	21(20)	20(19.2)
Married	83(79.8)	84(80.8)
Living arrangement n (%)		
With the patient		91 (87.5)
Not with the patient		13 (12.5)
Total time with the patient (hours)	21.72±5.41	21.72±5.41
Kinship to the patient n (%)		
Spouses: wife/husband		59(56.7)
Adult children: son/daughter		28(26.9)
Parents: mother/father		7(6.7)
Siblings		5(4.8)
Other family members		5(4.8)

* SD = Standard Deviation

The MAS scores were 1.14±1.05 for the upper limbs and 1.21±0.94 for the lower limbs in the spasticity assessment, which was based in a physical examination of the patient, corresponded to 3.63±1.70 for the arms, 3.16±1.87 for the hands and 3.89 ± 1.17 for the lower limbs. The FAS, FIM and SIS patient scores were 3.25±1.55, 87.11±28.92 and 57.20±18.17, respectively.

**Figure 1 -:**
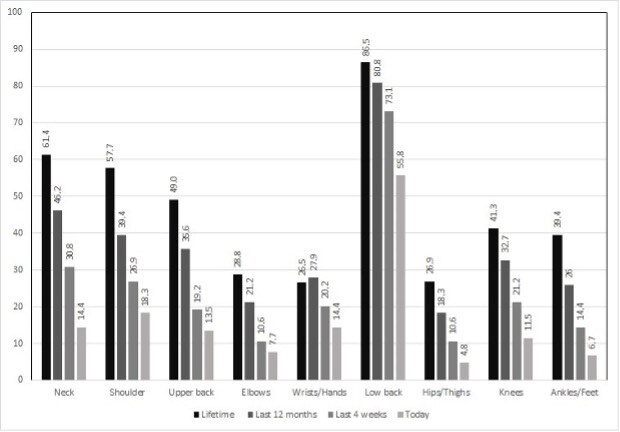
Prevalence of musculoskeletal symptoms by body area in the family caregivers. Istanbul, Turkey, 2019-2021

The Nordic Lifetime, 12-month, 4-week, and current caregivers’ scores are shown in Figure 1. 

Low back pain was the most common complaint (73.1%): in the last month, the FAS, FIM, SIS and Brunnstrom arm and leg scores were lower in caregivers with low back pain ( Table 2). Neck pain was the second most common musculoskeletal complaint after low back pain, whereas neck complaints were not statistically associated with any patient-related factors ( Table 2). 

Upon examination of complaints relating to the upper extremities, shoulder problems were associated with FAS, FIM, SIS, Brunnstrom scores for arms, hands and legs, MAS for upper and lower extremities, with a statistically significant difference observed in patient assessments related to stroke.

In caregivers with hand/wrist complaints, they were found to be related to MAS upper and lower limb patients’ scores ( Table 2). When lower limb problems were assessed, hip, knee, and foot pain was unrelated to patients who had ongoing care: complaints were related to the caregivers’ advanced age and high Body Mass Index (BMI) (p<0.05) ( Table 2 and Table 3). The BDS score was significantly higher in caregivers with low back, neck or upper back pain, as well as upper limb pain, except for wrist, knee and foot pain. The CBI score was also significantly higher in caregivers with low back pain, neck or upper back, upper limb and foot pain ( Table 3). 

**Table 2 -:** Caregivers’ musculoskeletal problems in relation to the stroke patients’ physical condition. Istanbul, Turkey, 2019-2021

	**Patients’ Age**	Patients’ BMI *	**FAS** † **Score**	**FIM** ‡ **Total**	**SIS** § **Total**	**Brunnstrom Stage Arm**	**Brunnstrom Stage Hand**	**Brunnstrom Stage Leg**	**MAS** ║ **Upper Limb**	**MAS** ║ **Lower Limb**
Low Back Yes n=76 No n=28 *p*	63.19±11.99 61.85±12.28 *0.587*	27.95±3.34 27.67±3.93 *0.541*	3.01±1.59 3.92±1.21 ***0.009***	82.51±28.99 99.60±25.21 ***0.005***	55.19±16.96 64.40±19.92 ***0.033***	3.43±1.80 4.17±1.24 ***0.040***	2.97±1.93 3.67±1.61 *0.127*	3.76±1.20 4.25±1.00 ***0.023***	1.19±1.10 1.00±090 *0.449*	1.25±0.98 1.10±0.83 *0.538*
Neck Yes n=32 No n=72 *p*	62.78±11.74 62.86±12.23 *0.924*	26.81±2.84 28.30±3.99 *0.090*	3.03±1.57 3.36±1.54 *0.341*	84.31±32.88 88.36±27.13 *0.559*	55.13±17.81 58.80±18.34 *0.382*	3.46±1.62 3.70±1.73 *0.522*	2.90±1.90 3.27±1.86 *0.226*	3.78±1.03 3.94±1.23 *0.296*	1.25±1.13 1.09±1.02 *0.534*	1.35±1.14 1.15±0.85 *0.482*
Shoulder Yes n=28 No n=76 *p*	65.60±12.22 61.81±11.87 *0.271*	28.57±4.28 27.45±3.55 *0.387*	2.71±1.38 3.46±1.57 ***0.019***	73.71±25.50 92.05±28.68 ***0.005***	50.17±11.87 60.43±19.34 ***0.005***	3.00±1.41 3.86±1.74 ***0.022***	2.21±1.47 3.51±1.89 ***0.001***	3.46±0.74 4.05±1.26 ***0.005***	1.67±1.09 0.94±0.97 ***0.002***	1.75±1.00 1.01±0.84 ***0.001***
Knee Yes n=22 No n=82 *p*	63.27±12.49 61.09±10.06 *0.262*	27.77±3.64 27.67±4.33 *0.689*	3.00±1.34 3.32±1.60 *0.277*	82.95±28.02 88.23±29.22 *0.428*	54.11±14.88 58.57±18.89 *0.306*	3.66±1.59 3.62±1.73 *0.967*	2.76±1.81 3.26±1.88 *0.163*	3.76±0.83 3.92±1.24 *0.410*	1.33±1.23 1.09±1.00 *0.413*	1.47±1.07 1.14±0.90 *0.195*
Wrist/Hand Yes n=21 No n=83 *p*	63.61±9.45 62.63±12.63 *0.703*	27.26±3.08 27.87±3.93 *0.574*	2.90±1.78 3.34±1.45 *0.377*	82.76±28.41 88.21±29.11 *0.402*	52.93±13.50 58.87±19.06 *0.228*	3.23±1.41 3.73±1.76 *0.272*	2.76±1.92 3.26±1.86 *0.181*	3.52±1.20 3.98±1.15 *0.104*	1.61±1.02 1.02±1.03 ***0.022***	1.71±0.95 1.08±0.90 ***0.008***
Upper back Yes n=20 No n=84 *p*	65.75±11.75 62.14±12.05 *0.225*	26.55±3.76 28.03±3.74 *0.104*	3.25±1.65 3.26±1.53 *0.909*	89.90±35.36 86.45±27.38 *0.473*	14.40±6.92 12.80±5.88 *0.428*	3.25±1.86 3.72±1.65 *0.256*	3.00±2.02 3.20±1.84 *0.504*	4.05±1.09 4.00±1.19 *0.758*	1.20±1.15 1.31±1.03 *0.824*	1.35±1.08 1.18±0.91 *0.441*
Foot/Ankle Yes n=15 No n=89 *p*	63.80±11.47 62.67±12.17 *0.875*	27.42±4.15 27.80±3.72 *0.614*	3.00±1.51 3.30±1.56 *0.495*	84.53±27.50 87.55±29.28 *0.708*	58.09±14.92 57.60±18.74 *0.778*	3.46±1.72 3.66±1.70 *0.696*	3.20±1.93 3.15±1.87 *0.820*	3.53±1.40 3.95±1.12 *0.271*	1.26±1.09 1.12±1.05 *0.631*	1.53±0.99 1.15±0.93 *0.161*
Elbow Yes n=11 No n=93 *p*	63.36±12.93 62.77±11.99 *0.941*	26.31±3.24 28.14±3.74 *0.068*	2.54±1.21 3.34±1.57 *0.085*	75.09±28.10 88.53±28.83 *0.152*	53.22±15.43 58.20±18.47 *0.509*	3.18±2.13 3.68±1.64 *0.337*	2.45±2.01 3.24±1.85 *0.123*	3.63±0.80 3.92±1.20 *0.305*	1.63±1.28 1.08±1.01 *0.127*	1.72±1.27 1.15±0.88 *0.089*
Hip Yes n=11 No n=93 *p*	63.71±12.67 62.42±12.73 *0.904*	29.24±4.53 27.57±3.66 *0.224*	3.00±1.67 3.29±1.54 *0.588*	86.45±35.18 87.19±28.32 *0.853*	61.71±23.77 57.19±17.50 *0.700*	4.27±1.90 3.55±1.67 *0.180*	3.90±1.81 3.07±1.81 *0.201*	4.09±1.51 3.87±1.13 *0.486*	0.72±0.78 1.19±1.07 *0.181*	1.00±0.89 1.23±0.95 *0.455*

* BMI = Body Mass Index;

† FAS = Functional Ambulation Score;

‡ FIM = Functional Independence Measure;

§ SIS = Stroke Impact Scale;

║ MAS = Modified Ashworth Scale

**Table 3 -:** Caregivers’ musculoskeletal problems in relation to their physical and emotional status. Istanbul, Turkey, 2019-2021

	**Caregiver’s Age**	Caregiver’s BMI *	**BECK Scores**	**CBI** † **Time Burden**	**CBI** † **Developmental**	**CBI** † **Physical**	**CBI** † **Emotional**	**CBI** † **Social**	**CBI** † **Total**
Low Back Yes n=76 No n=28 *p*	51.68±9.85 50.89±10.58 *0.651*	27.65±4.73 26.81±4.55 *0.531*	13.55±12.55 7.21±7.44 ***0.018***	12.31±5,92 7.03±6.20 ***<0.001***	7.05±6.36 4.14±4.24 *0.062*	6.03±5.17 3.89±4.90 ***0.029***	3.81±4.11 3.00±3.84 *0.297*	3.03±4.33 1.10±1.89 *0.085*	32.35±21.01 19.21±15.41 ***0.003***
Neck Yes n=32 No n=72 *p*	53.62±8.33 50.38±10.28 *0.176*	27.33±4.14 26.91±4.80 *0.428*	18.46±12.86 8.81±9.92 ***<0.001***	11.90±6.77 10.44±6.25 *0.275*	8.34±6.37 5.34±5.61 ***0.021***	8.56±4.77 4.08±4.75 ***<0.001***	4.87±4.64 3.02±3.63 *0.086*	3.28±3.78 2.18±3.95 ***0.041***	37.03±19.40 25.18±19.94 ***0.004***
Shoulder Yes n=28 No n=76 *p*	51.39±10.09 51.38±9.76 *0.863*	27.77±4.55 26.76±4.60 *0.365*	14.53±10.44 10.77±12.08 ***0.036***	13.96±5.41 9.76±6.42 ***0.002***	7.82±4.83 5.13±5.68 ***<0.001***	7.82±4.83 4.59±5.04 ***<0.001***	4.17±3.61 3.38±4.18 *0.130*	3.46±4.59 2.17±3.61 *0.075*	38.78±18.71 25.15±19.92 ***0.002***
Knee Yes n=22 No n=82 *p*	54.77±9.68 50.47±9.69 ***0.046***	29.51±4.71 26.61±4.01 ***0.016***	19.77±13.64 9.64±10.24 ***0.001***	11.45±7.55 10.74±6.12 *0.531*	8.18±6.84 5.75±5.67 *0.151*	8.68±5.97 4.59±4.59 ***0.006***	5.09±5.13 3.19±3.62 *0.146*	3.50±4.83 2.25±3.62 *0.299*	36.90±25.41 26.65±18.47 *0.124*
Wrist/Hand Yes n=21 No n=83 *p*	55.57±8.78 50.32±9.81 ***0.024***	27.69±5.04 26.87±4.49 *0.725*	18.42±11.55 10.10±11.23 ***0.002***	12.80±5.22 10.40±6.62 *0.183*	8.19±5.30 5.78±6.08 *0.051*	6.38±4.69 5.22±5.28 *0.167*	5.14±3.86 3.20±4.01 ***0.026***	3.04±3.27 2.38±4.07 *0.233*	35.61±16.85 27.10±20.99 ***0.037***
Upper back Yes n=20 No n=84 *p*	51.10±9.35 51.45±9.95 *0.782*	27.70±4.97 26.88±4.51 *0.695*	17.45±13.44 10.44±10.95 ***0.036***	10.30±7.30 11.03±6.23 *0.608*	8.30±5.99 5.78±5.92 *0.083*	7.40±4.33 5.00±5.26 ***0.029***	5.55±4.53 3.13±3.79 ***0.022***	4.35±4.72 2.08±3.60 ***0.006***	36.25±18.96 27.05±20.48 ***0.046***
Foot/Ankle Yes n=15 No n=89 *p*	57.13±7.16 50.41±9.88 ***0.011***	29.80±4.94 26.57±4.39 ***0.021***	17.46±13.23 10.83±11.26 ***0.044***	13.93±7.46 10.38±6.12 ***0.030***	9.33±5.39 5.75±5.96 ***0.027***	7.26±4.97 5.15±5.16 *0.124*	5.00±4.37 3.35±3.95 *0.129*	2.13±2.82 2.58±4.08 *0.980*	37.66±16.58 27.33±20.72 ***0.046***
Elbow Yes n=11 No n=93 *p*	54.09±11.05 51.06±9.65 *0.240*	28.43±7.29 26.87±4.19 *0.109*	18.36±14.53 11.01±11.20 *0.225*	14.54±5.78 10.46±6.38 ***0.041***	10.18±7.76 5.80±5.62 *0.089*	8.09±6.33 5.15±4.96 *0.179*	5.36±3.38 3.38±3.93 *0.155*	5.36±4.65 2.18±3.71 ***0.035***	43.54±26.31 27.08±19.06 ***0.036***
Hip Yes n=11 No n=93 *p*	58.09±7.62 50.59±9.75 ***0.010***	31.20±5.44 26.37±4.25 ***0.012***	12.45±12.04 11.70±11.76 *0.641*	14.18±7.20 10.50±6.24 ***0.042***	8.27±6.27 6.03±5.94 *0.199*	6.18±5.41 5.37±5.16 *0.616*	2.90±2.25 3.67±4.20 *0.965*	1.63±2.33 2.62±4.06 *0.770*	33.18±18.48 28.31±20.68 *0.395*

* BMI = Body Mass Index;

† CBI = Caregiver Burden Inventory

## Discussion

This study, pioneer in the English literature, associated caregivers’ complaints to functional abilities and disability degree in the stroke patients for whom they were responsible. As mentioned, our main objective was to determine specific symptoms in the caregivers, connect them to the conditions of the patients and determine areas where the caregivers were at risk, using precautions to avoid them.

Most of the caregivers in the current study belonged to the female gender. Women are usually required to perform caregiving tasks in the Turkish culture, as is the case in most Asian and Latin American populations ^(^ 6 ^,^ 24 ^-^ 25 ^)^. This might be attributed to social structures of Asian nations, where women are typically expected to care for ailing family members, as *per* social and cultural norms ^(^ 26 ^)^. Caregivers tend to be the patients’ spouses or children, coinciding with society’s framework that places the family at its center. Caregiving for stroke victims with high functional dependence was related to poorer physical and emotional health in the caregivers, as per recent literature analyses ^(^ 24 ^-^ 27 ^)^. We concluded that the caregivers’ health and patients’ functional state were strongly connected. 

Low back complaints dominated research involving nurses, physiotherapists and informal caregivers of people with impairments ^(^ 7 ^)^. As stated, low back pain was the most common complaint among the caregivers, which was consistent with the literature and related to the patients’ FAS, FIM, SIS and Brunnstrom scores. Another study revealed that 82.8% of the caregivers of stroke patients reported low back pain ^(^ 9 ^)^. The low back is stressed by lifting, transfers and assistance with daily tasks. Caring for stroke patients with high functional dependence levels represents an additional physical effort, which can lead to stress in caregivers ^(^ 28 ^-^ 29 ^)^. No relation was found between the patients’ MAS scores and low back pain. Spasticity may result in an extensor synergistic activation pattern in the lower limbs while standing and walking. In turn, this may ease walking by locking the hip and knee joints in their extended position, in addition to supporting independence of the patients by aiding ambulation. Absence of a relationship between low back pain and spasticity can be explained by the fact that spasticity is a factor that helps ambulation ^(^ 30 ^)^. 

Common complaints among the caregivers were neck and upper back pain. Neck pain was their second most common complaint; however, with upper back pain, neck pain was not associated with any of the patient functional parameter as related to the stroke. Shoulders and upper limbs were the most affected body areas for the caregiving activity. Our study showed that upper limb complaints were significantly linked to all the patients’ evaluations related to the stroke. The shoulders, cervical region, arms, lumbar region and lower limbs were the most common sites for musculoskeletal problems in caregivers aged between 25 and 60 years old, which is consistent with the study findings ^(^ 31 ^)^. In addition, hand and wrist complaints were exclusively related to MAS upper and lower limb patients’ scores. As interpreted from these results, upper limb problems were related to patient spasticity. In applications that required resistance during patient manipulation, problems could occur in the shoulder and wrist joints, relatively more mobile and, thus, weaker. In this study, hip-related and lower limb complaints did not seem to have any relation to the physical condition of the patient; instead, they were related to the caregivers’ age and BMI. 

The risk of musculoskeletal problems among the caregivers increased with lack of appropriate training and instruction in the use of optimal techniques. According to a study, 94.4% never underwent training on how to properly attend to stroke patients ^(^ 7 ^)^. Other literature materials revealed that stroke caregivers lacked this kind of education ^(^ 32 ^)^. 

The literature shows the caregivers’ high burden, plus the enormous physical strain on them. As such, extensive care impacted their physical health. The symptoms might not be related to patient care, but burden depression were perceived by the patients’ relatives. In this study, caregivers with neck and upper back pain had significantly higher depression levels. According to a comprehensive study, depression, muscle tension and perceived role conflict were the main psychosocial risks for neck discomfort in the literature ^(^ 33 ^)^. In a study of caregivers of patients with chronic neurological disorders, it was discovered that the physical and mental care burden was inversely related to patients’ functional independence ^(^ 34 ^)^. These results may be the natural effects of aging and weight on the musculoskeletal system, with lower limbs affected the most. Hip and lower limb complaints did not seem to increase the CBI scores, which may be explained by caregivers not attributing these symptoms to their workload, but to their own physical condition. However, all caregiver complaints included neck, shoulders, back and upper limbs, increasing both their burden and depression levels. In general, it was found that the patients’ age and BMI did not affect the caregivers’ Nordic scores. The main challenge for them was the patients’ functional capacity, rather than their weight. We might add that the fact that a patient was not overweight did not tend to have a protective effect, as caregivers dealing with thin patients had similar complaints (and at the same rate) as those dealing with overweight patients. 

According to the findings of this study, the low back was commonly impacted by musculoskeletal complaints in family caregivers. The study results also showed a high correlation between the patients’ functional capacity levels and the caregivers’ musculoskeletal problems. Providing physical and psychological assistance to family caregivers has become a social issue. The frequency of caregivers’ musculoskeletal problems and their patients’ needs must be addressed, as training should become an integral aspect of rehabilitation programs.

Representatives of the public health system, including nurses and other health professionals, must ensure that providing care for a family member does not lead to a negative impact ^(^ 35 ^)^. In this context, caregiver education is critical. Health professionals must be aware of the family caregivers to determine overall success ^(^ 36 ^)^. In addition, most nurses are women and, combined with their professional caregiving skills, many became family caregivers. Studies from other countries show different approaches to integrating the family perspective in Nursing education. If family care is not included, they may not develop as caregivers in patient-centered teams, or meet their own health needs ^(^ 37 ^-^ 38 ^)^. The data explain musculoskeletal problems that may be experienced by caregivers, the frequency of these problems, and their relationship to the patients’ condition. 

A number of studies have shown that the prevalence of chronic pain in aged patients is around 40% ^(^ 39 ^)^. In our study, we aimed at determining the relationship with presence of musculoskeletal pain in the caregivers; therefore, patients over the age of 65 were not included in order to rule out the degenerative process and the chronic pain it may cause. The fact that we excluded patients over the age of 65 may have prevented us from detecting the problems of aged caregivers, which is an important limitation of our study. 

Another limitation of this study is that it follows a cross-sectional design and that it cannot be clarified whether these problems are exactly related to care. In our study, the patients’ self-reported pain was obtained by means of a questionnaire method. Another study limitation is the absence of diagnoses for complaints by physical examination and imaging methods. In turn, one of its strengths is having excluded aged caregivers, who have more frequent musculoskeletal problems. When examining the data of this study, it should be kept in mind that there may be bias due to the study design. Consequently, any additional generalizations drawn from this study should be used with caution.

## Conclusion

The results of our study can be valuable in terms of preventive medicine, which should become part of nursing education. This study showed that musculoskeletal symptoms are highly prevalent among family caregivers of stroke survivors, with the low back as the most affected body area. The study findings also indicate that musculoskeletal symptoms are strongly related to the functional capacity level of the patient. As providing physical and psychological assistance to family caregivers is becoming a social issue, it is vital to look into their current circumstances. The high frequency of musculoskeletal problems among caregivers and their specific complaints in regard to the special and unique needs of the patient should be addressed, and education and training of stroke caregivers should be incorporated into rehabilitation programs.
